# Optimal design of experiments for functional linear models with dynamic factors

**DOI:** 10.1007/s11749-026-01004-z

**Published:** 2026-03-12

**Authors:** Caterina May, Theodoros Ladas, Davide Pigoli, Kalliopi Mylona

**Affiliations:** 1 Department of Mathematics, King’s College London, Strand Campus, London, WC2R 2LS UK; 2 Dipartimento DiSEI, Università del Piemonte Orientale, V. Ettore Perrone 18, 28100 Novara, Italy

**Keywords:** Dynamic experimental conditions, Estimation, Function-on-function linear model, Functional data analysis, Optimal design of experiments, 62K05, 62R10

## Abstract

In this work, we build optimal experimental designs for precise estimation of the functional coefficient of linear regression model where both the response and the factors are continuous functions. After obtaining the variance–covariance matrix of the estimator of the functional coefficient which minimizes the integrated sum of square of errors, we extend the classical definition of optimal design to this estimator, and we provide the expression of the A-optimal and of the D-optimal designs. Examples of optimal designs for dynamic experimental factors are then computed through a suitable algorithm, and we discuss different scenarios in terms of the set of basis functions used for their representation. Finally, we present an illustrative example inspired by a real application in a pharmaceutical manufacturing process, to illustrate the feasibility and the advantages of our methodology.

## Introduction

In many applications, data are collected as curves over time, and it is therefore natural to consider statistical models with curves (or functions) as response and as covariates. This kind of problems has been considered for example in areas such as biomedical sciences (Shi et al. 2012), genetics (Müller et al. 2008), pharmaceutical sciences (Rahman et al. 2019) and transport engineering (Zhang et al. 2018), to mention just a few. However, most of these works have been concerned with observational data and little attention has been devoted to experimental design for settings where both the covariates and the response are functional in nature. An example of a laboratory experiment that is within this framework and motivated this research is the one described in Pigoli et al. (2023), in the context of forensic entomology. Entomologists are interested in studying the relationship between larval growth (and growth rate) and temperature, but both these quantities vary in time. In a laboratory setting, the current practice is to keep the incubator temperature constant for each experimental run, and then having various runs at different temperatures. However, this is a choice based only on practicality, since it does not require the operator to change the incubator temperature during the run. A natural question is if there are better experimental designs to explore the relationship between the growth rate curve and the temperature curve. This is an example of the kind of scenarios we wish to address in this work. Another example is the ergonomic experiment discussed in Aletti et al. (2016a, 2016b) to forecast car driver’s arm motion. Here, the control variables are the spatial coordinates of a target object inside the car cabin, which are scalar. The functional response is the angle formed at the right elbow between the upper and the lower arm varying during time to reach this object. As an extension to functional control variables, the target locations can change to specific trajectories that the hand must follow to move an object from one position to another. Other examples arise in industrial settings, where the response is functional (e.g., near infra-red spectroscopy measurements in chemical or pharmaceutical manufacturing, Blanco et al. 1998) and the process parameters (such as temperature and speed) can be varied over time. More recently, Chen et al. (2021) illustrated an application in bioengineering, where function-on-function Gaussian process regression is used to emulate the tissue-mimicking performance of synthetic materials. A motivating example, inspired by the pharmaceutical industry, will be described in the next section.

One of the main tasks of optimal experimental design is the choice of the best experimental conditions to estimate the unknown parameters of a statistical model. D-optimality and A-optimality, studied in this paper, are popular criteria that aim to minimize the generalized variance and the average variance of the parameter estimators, respectively. Classical references on optimal experimental design are, for instance, Fedorov (1972), Silvey (1980), Pukelsheim (2006) and Atkinson et al. (2007). The use of optimal design is crucial, since it can drastically lower the cost of experimentation by reducing the total number of observations needed to guarantee the desired precision in parameter estimation.

We consider a framework where both the experimental factors and the responses have a functional nature. In practice, the data analysis always starts from raw measurements, and a preprocessing smoothing step is required to represent observations as functions. We refer to Ramsay and Silverman (2005) for an extensive discussion of the smoothing procedures that can be used to reconstruct the functional data; the starting point of our work will be responses and factors that are already represented as functions. Also, we restrict ourselves to the case of *densely observed* functional data, (Gertheiss et al. 2024), where smoothing can indeed be treated as a preprocessing step, while we will not discuss the case of *sparsely observed* functional data, where the location of the measurements can pose in itself an experimental design issue (see, e.g., Rha et al. 2020; Kao and Huang 2024).

In terms of statistical models, in this work we will consider function-on-function linear models (see, e.g., Ramsay and Silverman (2005) or Horváth and Kokoszka (2012)). To the best of our knowledge, statistical inference on these models has not received much attention. The original contribution of this paper is therefore twofold. First, we derive the expression of the covariance of the estimator obtained after basis expansion of the functional coefficient (Ramsay and Silverman 2005). Second, we use this result to construct optimal experimental designs with dynamic factors, which guarantees the most precise estimation. This will expand the current state of the art on experimental design for functional data, where optimal designs for experiments for scalar-on-function linear models (i.e., models with functional covariates but scalar response) have been considered by Michaelides et al. (2023) and for function-on-scalar linear models have been discussed in Aletti et al. (2014) and Aletti et al. (2016b). On the other hand, previous work on optimal design of experiment for problems with time-varying factors and response (see, e.g., Fedorov and Hackl 1997) has focused on models in which the functional form of the relationship between factors and responses is known (e.g., quadratic) or on kriging estimators (Morris 2014). In our approach, we instead consider the functional data framework where the model coefficients are themselves functional objects, represented in a flexible way through a basis expansion.

The paper is organized as follows. Section 2 describes in detail an illustrative example. The model is presented in Sect. 3. In Sect. 4, the estimator of the functional coefficient of multiple factors is derived, and its properties are proved. Section 5 is devoted to the construction of exact optimal experimental design on the base of the inference results previously obtained. In addition, Sect. 6 illustrates and discusses some concrete examples of optimal designs, obtained with an optimization algorithm. In Sect. 7, inspired by the motivating example in Sect. 2, we present a proof-of-concept example with simulated data, as an illustration of our methodology for practitioners. In Sect. 8, conclusions and future developments are discussed.

## Motivating example

We illustrate the applicability of our framework with an industrial scenario based on a pharmaceutical manufacturing process (Van Snick et al. 2019). The process involves feeding material into a continuous line through a gravimetric feeder, while the outlet concentration of the material is measured in-line downstream. Subsequently, the mixture of material is abruptly changed, and it is measured how the resulting outlet concentration changes over the time of the experimental run.

Hence, each experimental run can be viewed as a dynamic input–output system. The input is the dosing program applied at the feeder, which specifies the exact mixture of material that is fed over time. In practice, these dosing profiles are typically stepwise functions, as the feeder may be turned on or off, or operated at a few different levels in successive time intervals. The output is the concentration measured at the outlet, also observed over time. Because of transport delays and mixing, changes in the input are reflected in the output only after some lag, and the response tends to be smoother than the input. This leads to a setting where both the experimental factors and the responses are functions of time. Each run corresponds to one pair of functional trajectories: a dosing profile and the resulting concentration curve.

In the original study of Van Snick et al. (2019), these functional quantities were simplified into scalar or low-dimensional summaries before designing the experiment. For instance, researchers worked with cumulative or normalized versions of the concentration curve (such as residence-time distributions). The experiment was then designed for these scalar parameters rather than for the underlying functional relationship.

Our framework treats the dosing program and the measured concentration directly as functions. Instead of designing an experiment around scalar summaries, we design the specific trajectories of mixture of material to be tested. The goal is to select a small number of profiles that provide the most precise information about how input activity at one time influences the response at another time.

**Fig. 1:**
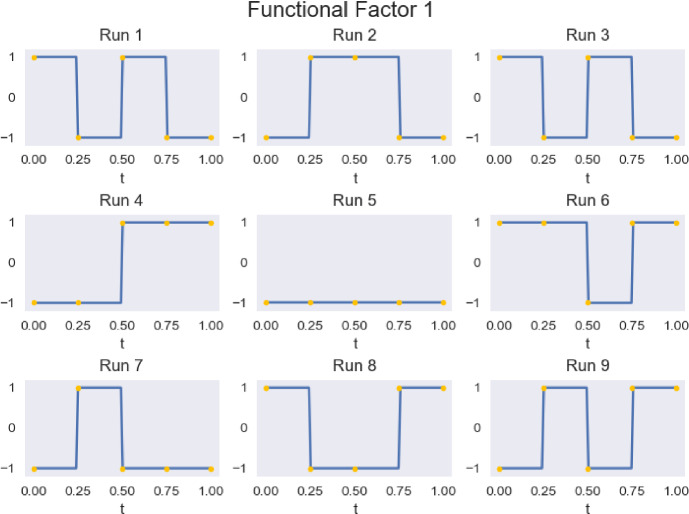
Example of a nine-run A-optimal design for the industrial case study. Each panel shows the dosing trajectory of a single experimental run as a stepwise input over normalized time t∈[0,1]

A possible A-optimal design with 9 runs obtained under our framework is illustrated in Fig. 1. Each of the nine runs corresponds to a distinct dosing program applied to the feeder, where the feed level switches between -1 (lowest setting) and 1 (highest setting) at different time intervals. These stepwise profiles were obtained using a step-function basis for the input factor, reflecting the discrete operating modes of the feeder. The corresponding process response was represented using a smoother, second-order spline basis to capture the gradual transport and mixing effects observed in the outlet concentration. This combination of stepwise input and smooth output is consistent with the physical behavior the experimental setup of Van Snick et al. (2019). Collectively, they provide rich coverage of the input domain, ensuring that changes in the input mixture occur at various stages of the run. This diversity allows the resulting data to capture how early, mid-run, and late changes in dosing propagate through the system.

## Model and notation

Throughout this paper, we consider the following function-on-function linear model, which generalizes to multiple factors the model introduced by Ramsay and Dalzell (1991) and further discussed, for example, by Ramsay and Silverman (2005) and Kokoszka and Reimherr (2017):

1 $$\begin{aligned} y_n(t)=\beta _0(t)+\sum _{i=1}^p\int _0^\mathcal{T} \beta _i(s,t)x_{ni}(s)\textrm{d}s + \varepsilon _n(t), \quad n=1, ...,N, \quad p\ge 1 \end{aligned}$$

where $y_n \in L^2([a,b]), x_{ni} \in L^2([0,\mathcal{T}], \beta _0\in L^2([a,b])$ and $\beta _i \in L^2 ([0, \mathcal{T}] \times [a,b])$ are square-integrable functions, and $\varepsilon _1(t), \ldots , \varepsilon _N(t)$ are i.i.d. zero-mean stochastic processes (not necessarily Gaussian). Without loss of generality, we assume that all factors are defined over the same interval $[0, \mathcal{T} ]$. If this is not the case—i.e., if the factors are defined on different intervals or involve different independent variables such as time, frequency, or spatial coordinates—they can nevertheless be rescaled to a common standardized interval $[0, \mathcal{T} ]$. Similarly, the independent variable *t* in the response is not necessarily the same as in the factors and even when it is the same, it can be defined on a different interval.

Furthermore, the model formulation in (1) assumes that the response at any point may depend on the entire domain of the factor. In the special case where the independent variable is time for both the response and one or more of the factors, this assumption may be unrealistic, as it would imply that the response depends on future values of the factor. In such cases, a more appropriate approach is the *historical functional linear model* (Malfait and Ramsay 2003), in which the integration is restricted to the interval [0, *t*]. While the general methodology proposed in this paper can be extended to the historical model, it requires additional numerical computations to approximate the integrals. For this reason, we do not consider the historical model further in this work.

In matrix notation, model (1) can be rewritten as

2 $$\begin{aligned} \textbf{y}(t)=\int _0^\mathcal{T} \mathcal {X}(s) {\pmb {\beta }}(s,t)\textrm{d}s + {\pmb {\varepsilon }}(t), \end{aligned}$$

where $\textbf{y}(t)=(y_1(t), \ldots , y_N(t))^T$ is the vector of responses, $\pmb {\varepsilon }(t)=(\varepsilon _1(t), \ldots , \varepsilon _N(t))^T$ is the vector of errors, $\pmb {\beta }(s,t)=(\beta _0(t), \beta _1(s,t), \ldots , \beta _p(s,t))^T$ is the functional coefficient to be estimated, and $\mathcal{X}(s)$ is the N×(p+1) model matrix:

3 $$\begin{aligned} \mathcal {X}(s)=[\textbf{1}_N \,|\,\textrm{X}(s)], \end{aligned}$$

with $\textrm{X}(s)$ being the N×p design matrix of the dynamic predictors:

4 $$\begin{aligned} \textrm{X}(s)= \begin{bmatrix} \textbf{x}_1(s)&\textbf{x}_2(s)&\ldots&\textbf{x}_p(s) \end{bmatrix} =\begin{bmatrix} x_{11}(s) & \ldots & x_{1p}(s) \\ x_{21}(s) & \ldots & x_{2p}(s) \\ \vdots & & \vdots \\ x_{N1}(s) & \ldots & x_{Np}(s) \\ \end{bmatrix} \end{aligned}$$

We consider a basis $\{\theta _l(t), l\ge 1\}$ to expand the responses $y_n(t)$, and the errors $\varepsilon _n(t)$, for n=1,...,N. We can then expand the intercept $\beta _0(t)$ and the kernels $\beta _i(s,t)$, for any i=1,...,p, according to the basis $\{\theta _l(t), l\ge 1\}$ and to *p* bases $\{\eta ^1_k(s), k\ge 1\}$,..., $\{\eta ^p_k(s), k\ge 1\}$:

$$\begin{aligned} \beta _0(t)=\sum _l b^0_l\, \theta _l(t), \end{aligned}$$

$$\begin{aligned} \beta _i(s,t)= \sum _{k,l} b^i_{k,l}\, \eta ^i_k(s)\, \theta _l(t). \end{aligned}$$

Since, by the Gram–Schmidt orthonormalization procedure, any basis can be transformed into an orthonormal basis, from now on we assume that the basis $\{\theta _l(t), l\ge 1\}$ is orthonormal, that is,

$$\begin{aligned} \langle \theta _h(t), \theta _k(t) \rangle _{L^2}= \int \theta _h(t) \theta _k(t) \textrm{d}t = \delta _{hk}, \end{aligned}$$

where is the Kronecker delta symbol: $\delta _{hk}=1$ if h=k and $\delta _{hk}=0$ if h≠k.

In order to estimate the parameters of the functional model (1), we follow the ideas contained in Ramsay and Silverman (2005) and in Horváth and Kokoszka (2012); their fully functional model is here extended to multiple factors and, in Sect. 4, we derive explicitly the estimator for the parameters of our model. Let us project the unknown coefficients in the subspaces generated by the finite bases $\{\theta _l(t), 1 \le l \le L\}$ and $\{\eta ^1_k(s), 1 \le k\le K^1\}$,..., $\{\eta ^p_k(s), 1 \le k\le K^p\}$. Note that each coefficient $\beta _i(s,t)$ for i=1,...,p, can be expanded, with respect to their first variable *s*, in a different basis and with a proper dimension $K^i$, according to the features of the corresponding factor.

We have:

$$\begin{aligned} \beta _0^*(t)= {\textbf{b}^0}^T \, \pmb {\theta }(t), \end{aligned}$$

$$\begin{aligned} \beta _i^*(s,t)= \pmb {\eta }^i(s)^T \textrm{B}^i \, \pmb {\theta }(t), \end{aligned}$$

for any i=1,...,p, where

$$\begin{aligned} {\textbf{b}^0}=(b_1^0, \ldots , b_L^0)^T, \end{aligned}$$

$$\begin{aligned} \pmb {\theta }(t)=(\theta _1(t), \ldots , \theta _{L}(t))^T, \end{aligned}$$

$$\begin{aligned} \pmb {\eta }^i(s)=(\eta ^i_1(s), \ldots , \eta ^i_{K^i}(s))^T, \end{aligned}$$

and

$$\begin{aligned} \textrm{B}^i=[ b^i_{k,l}] \end{aligned}$$

is a $K^i \times L$ matrix. Then, the functional coefficient $\pmb {\beta }(s,t)$ becomes

5 $$\begin{aligned} \pmb {\beta }^*(s,t)=(\beta _0^*(t), \beta _1^*(s,t), \ldots , \beta _p^*(s,t))^T = \textrm{H}(s)^T \, \textrm{B}\, \pmb {\theta }(t) \end{aligned}$$

where $\textrm{H}(s)^T$ is a $(p+1)\times (1+K^1+...+K^p)$ diagonal block matrix given by

$$\begin{aligned} \textrm{H}(s)^T=\text{ diag }(1, \pmb {\eta }^1(s)^T, \ldots ,\, \pmb {\eta }^p(s)^T), \end{aligned}$$

and $\textrm{B}$ is an $(1+K^1+...+K^p) \times L$ block matrix of coefficients

$$\begin{aligned} \textrm{B}=\begin{bmatrix} {\textbf{b}^0}^T\\ \textrm{B}^1\\ \vdots \\ \textrm{B}^p \end{bmatrix} \end{aligned}$$

to be estimated. Model (2) becomes:

$$\begin{aligned} \textbf{y}(t)= \int _0^\mathcal{T} \mathcal {X}(s) \pmb {\beta }^*(s,t)\textrm{d}s + {\pmb {\varepsilon }}(t), \end{aligned}$$

that is, putting the (5) into it,

6 $$\begin{aligned} \textbf{y}(t)=\textrm{Z} \, \textrm{B}\, \pmb {\theta }(t) + \pmb {\varepsilon }(t), \end{aligned}$$

where $\textrm{Z}$ is the $N \times (1+K^1+...+K^p)$ matrix given by

7 $$\begin{aligned} \textrm{Z}= \int \mathcal {X}(s) \textrm{H}(s)^T \,\textrm{d}s. \end{aligned}$$

## Estimation of the model

In this section, we derive an estimator of the unknown functional coefficients of model (3), and we obtain the expression of its variance. This is equivalent to estimating the unknown block matrix $\textrm{B}$ of coefficients in (6); to this aim, we minimize the Integrated Sum of Square of Errors (ISSE), and then, we define the matrix estimator

8 $$\begin{aligned} \hat{\textrm{B}}=\arg \min _{\textrm{B}} \text{ ISSE }, \end{aligned}$$

where

9 $$\begin{aligned} \text{ ISSE }= &  \int \Vert \textbf{y}(t) - \textrm{Z} \, \textrm{B}\, \pmb {\theta }(t) \Vert ^2 \textrm{d}t \nonumber \\= &  \int {{\,\textrm{tr}\,}}(\textbf{y}(t) - \textrm{Z} \, \textrm{B}\, \pmb {\theta }(t))^T (\textbf{y}(t) - \textrm{Z} \, \textrm{B}\, \pmb {\theta }(t)) \textrm{d}t \nonumber \\= &  {{\,\textrm{tr}\,}}\int (\textbf{y}(t)^T\textbf{y}(t) + \pmb {\theta }(t)^T \textrm{B}^T\, \textrm{Z}^T\, \textrm{Z}\, \textrm{B}\, \pmb {\theta }(t) - 2 \pmb {\theta }(t)^T \textrm{B}^T\, \textrm{Z}^T\, \textbf{y}(t)) \textrm{d}t \nonumber \\= &  {{\,\textrm{tr}\,}}\int \textbf{y}(t)^T\textbf{y}(t) dt + {{\,\textrm{tr}\,}}\textrm{B}^T\, \textrm{Z}^T\, \textrm{Z}\, \textrm{B}\, \textrm{J}_{{\theta }{\theta }} - 2 {{\,\textrm{tr}\,}}\textrm{B}^T\, \textrm{Z}^T\, \int \textbf{y}(t)\, \pmb {\theta }(t)^T \textrm{d}t ; \end{aligned}$$

the last equality follows from the cyclic property of the trace and by defining

$$\begin{aligned} \textrm{J}_{{\theta }{\theta }}=\int \pmb {\theta }(t)\, \pmb {\theta }(t)^T\, \textrm{d}t. \end{aligned}$$

To obtain the estimator $\hat{\textrm{B}}$, we calculate the matrix derivatives of (9) and set them equal to zero; according to the properties of matrix calculus we have:

$$\begin{aligned} \dfrac{\partial }{\partial \textrm{B}} {{\,\textrm{tr}\,}}\textrm{B}^T\, \textrm{Z}^T\, \textrm{Z}\, \textrm{B}\, \textrm{J}_{{\theta }{\theta }}- &  2 \dfrac{\partial }{\partial \textrm{B}} {{\,\textrm{tr}\,}}\textrm{B}^T\, \textrm{Z}^T\, \int \textbf{y}(t)\, \pmb {\theta }(t)^T \textrm{d}t\\= &  2\, \textrm{Z}^T\, \textrm{Z}\, \textrm{B}\, \textrm{J}_{{\theta }{\theta }} - 2\, \textrm{Z}^T\, \int \textbf{y}(t)\, \pmb {\theta }(t)^T \textrm{d}t = 0, \end{aligned}$$

and hence, we obtain the normal equations:

$$\begin{aligned} \textrm{Z}^T \textrm{Z}\, {\textrm{B}}\, \textrm{J}_{{\theta }{\theta }} = \textrm{Z}^T \int \textbf{y}(t) \, \pmb {\theta }(t)^T\, \textrm{d}t. \end{aligned}$$

Since the basis $\{\theta _l(t), l\ge 1\}$ is orthonormal, then $\textrm{J}_{{\theta }{\theta }}= \textrm{I}_L$; if $\textrm{Z}$ in (7) has maximum rank $(1+K^1+...+K^p)$, that is, $\textrm{Z}^T \textrm{Z}$ is invertible, we have the following expression of the estimator (8):

10 $$\begin{aligned} \hat{\textrm{B}} = (\textrm{Z}^T \textrm{Z})^{-1}\textrm{Z}^T \int \textbf{y}(t) \, \pmb {\theta }(t)^T\, \textrm{d}t. \end{aligned}$$

The following result provides the variance of the vectorization of the matrix estimator (10). Given a p×q matrix $\textrm{M}$, ${{\,\textrm{vec}\,}}(\textrm{M})$ is the *pq*-vector obtained by stacking the columns of $\textrm{M}$; the symbol ⊗ denotes the Kronecker product on two matrices.

### Proposition 1

The estimator (10) is an unbiased estimator of the matrix $\textrm{B}$ of coefficients of $\pmb {\beta }^*(s,t)$, and its variance is

$$\begin{aligned} Var({{\,\textrm{vec}\,}}{\hat{\textrm{B}}})= \mathrm {\Sigma } \otimes (\textrm{Z}^T \textrm{Z})^{-1}. \end{aligned}$$

where $\mathrm {\Sigma }$ is a L×L matrix whose elements are

$$\begin{aligned} \mathrm {\Sigma }_{il}=Cov\left( e_{ni}, e_{nl}\right) , \end{aligned}$$

not depending on *n*, with $e_{ni}$ and $e_{nl}$ being the *i*-th and the *l*-th coefficients, respectively, of the expansion of $\varepsilon _n(t)$ in the basis $\{\theta _l(t), l=1,..., L\}$.

### Proof

The unbiasedness follows from the interchangeability between expectation and integration, and because $\textrm{J}_{{\theta }{\theta }}= \textrm{I}_L$, from the orthonormality of $\{\theta _l(t), l=1,..., L\}$.

By considering the representation $\textbf{y}(t)= \textrm{Y} \pmb {\theta }(t)$ of the response vector, where $\textrm{Y}$ is a N×L matrix of coefficients, we have that

$$\begin{aligned} \int \textbf{y}(t) \, \pmb {\theta }(t)^T\, \textrm{d}t = \textrm{Y}\, \textrm{J}_{\theta \theta }=\textrm{Y}, \end{aligned}$$

and thus, the estimator (10) can be expressed as $\hat{\textrm{B}} = (\textrm{Z}^T \textrm{Z})^{-1}\textrm{Z}^T \textrm{Y}$. Note that the random coefficients in each *n*-th row of $\textrm{Y}$, for n=1,...,N, are dependent between them, since they expand the same functional response $y_n(t)$, while each row is independent on the others since each row expands a different response.

In order to obtain an expression of the variance of estimator (10), it is then convenient to concatenate the rows of $\textrm{Y}$ and, to get it, to vectorize its transpose ${\hat{\textrm{B}}}^T$:

$$\begin{aligned} {\hat{\textrm{B}}}^T = \textrm{Y}^T \textrm{Z}\,(\textrm{Z}^T \textrm{Z})^{-1} . \end{aligned}$$

If $\mathrm {\Phi }$ and $\mathrm {\Psi }$ are a p×q and an r×s matrices, respectively, it is possible to show that

$$\begin{aligned} {{\,\textrm{vec}\,}}(\mathrm {\Phi }\mathrm {\Psi })=(\mathrm {\Psi }^T\otimes \textrm{I}_p){{\,\textrm{vec}\,}}(\mathrm {\Phi }). \end{aligned}$$

Hence,

$$\begin{aligned} {{\,\textrm{vec}\,}}{{\hat{B}}}^T= [( (\textrm{Z}^T \textrm{Z})^{-1}\,\textrm{Z}^T)\otimes \textrm{I}_L ] {{\,\textrm{vec}\,}}(\textrm{Y}^T) \end{aligned}$$

and

$$\begin{aligned} Var({{\,\textrm{vec}\,}}{\hat{\textrm{B}}}^T)=\textrm{F} \, \mathrm {\Delta } \, \mathrm {F^T}, \end{aligned}$$

where

$$\begin{aligned} \textrm{F}=[( (\textrm{Z}^T \textrm{Z})^{-1}\, \textrm{Z}^T)\otimes \textrm{I}_L ] \quad \text{ and } \quad \mathrm {\Delta }=Var({{\,\textrm{vec}\,}}\textrm{Y}^T), \end{aligned}$$

which are, respectively, a $(1+\sum _{i=1}^p K^i)\, L \times NL$ and a NL×NL matrices. To obtain the matrix Σ, observe that since $y_1(t), \dots , y_N(t)$ are independent and identically distributed stochastic processes, then the columns of $\textrm{Y}^T$, that is, $\int y_1(t)\,\pmb {\theta }(t)^T\, \textrm{d}t,..., \int y_N(t)\,\pmb {\theta }(t)^T\, \textrm{d}t,$ are i.i.d. random vectors. Then,

$$\begin{aligned} \Delta = I_N \otimes \Sigma , \end{aligned}$$

where

11 $$\begin{aligned} \Sigma _{il}=Cov\left( \int y_n(t)\theta _i(t)\, \textrm{d}t, \int y_n(t)\theta _l(t)\, \textrm{d}t\right) \end{aligned}$$

which does not depend on *n*. As a consequence of model (1), the second term in (11) is equal to

$$\begin{aligned} Cov\left( \int \varepsilon _n(t)\theta _i(t)\, \textrm{d}t, \int \varepsilon _n(t)\theta _l(t)\, \textrm{d}t\right) ; \end{aligned}$$

moreover, for any *n* and *i*,

$$\begin{aligned} \int \varepsilon _n(t)\theta _i(t)\, \textrm{d}t = \int \sum _{l=1}^L e_{nl} \theta _l(t)\theta _i(t)\, \textrm{d}t = \sum _{l=1}^L e_{nl}\int \theta _l(t)\theta _i(t)\, \textrm{d}t = e_{ni}, \end{aligned}$$

where the last equation follows from the orthonormality of $\{\theta _l, l=1,..., L\}$. We finally obtain

12 $$\begin{aligned} Var({{\,\textrm{vec}\,}}{\hat{\textrm{B}^T}})= &  [( (\textrm{Z}^T \textrm{Z})^{-1}\textrm{Z}^T)\otimes \textrm{I}_L ]\, (\textrm{I}_N \otimes \mathrm {\Sigma })\,\nonumber [( \textrm{Z}\,(\textrm{Z}^T \textrm{Z})^{-1})\otimes \textrm{I}_L ] \\= &  (( (\textrm{Z}^T \textrm{Z})^{-1}\textrm{Z}^T) \otimes \mathrm {\Sigma })\, [( \textrm{Z}\,(\textrm{Z}^T \textrm{Z})^{-1})\otimes \textrm{I}_L ] \nonumber \\= &  (\textrm{Z}^T \textrm{Z})^{-1}\otimes \mathrm {\Sigma }. \end{aligned}$$

The two last equalities are due the following property of the Kronecker product: If $\textrm{A}$, Φ, Ψ and Ω are matrices of sizes that can form the matrices products $\textrm{A}\, \Psi$ and $\Phi \, \Omega$, then

$$\begin{aligned} (\textrm{A} \otimes \Phi )(\Psi \otimes \Omega )=(\textrm{A}\, \Psi )\otimes (\Phi \, \Omega ). \end{aligned}$$

Finally, to obtain $Var({{\,\textrm{vec}\,}}{\hat{\textrm{B}}})$ from the (12), let us denote by $K^{(p,q)}$ the commutation matrix of dimension pq×pq such that ${{\,\textrm{vec}\,}}(M^T)=K^{(p,q)}{{\,\textrm{vec}\,}}(M)$ for any p×q matrix *M* (see (Magnus and Neudecker (2019), Chapt. 18). Then,

$$\begin{aligned} {{\,\textrm{vec}\,}}{\hat{\textrm{B}}}=K^{(L,1+\sum _{i=1}^p K^i)}{{\,\textrm{vec}\,}}({\hat{\textrm{B}^T}}), \end{aligned}$$

and hence,

$$\begin{aligned} Var({{\,\textrm{vec}\,}}{\hat{\textrm{B}}})= &  {K^{(L,1+\sum _{i=1}^p K^i)}((\textrm{Z}^T \textrm{Z})^{-1}\otimes \mathrm {\Sigma })K^{(1+\sum _{i=1}^p K^i,L)}}\\= &  \mathrm {\Sigma } \otimes (\textrm{Z}^T \textrm{Z})^{-1}, \end{aligned}$$

where the first equality is justified by the property ${K^{(p,q)}}^T=K^{(q,p)}$, while the final equality follows from the property of the commutation matrices with Kronecker product (see again Magnus and Neudecker (2019, Chapt. 18)). □

## Optimal experimental designs

An experimental design for the model described in Sect. 3 is defined as a set of experimental conditions $x_{n1}(s), \dots , x_{np}(s)$ in the interval $[0,\mathcal{T}]$ chosen by the experimenter for each experimental run n=1,⋯,N, according to a specific experimental objective; an experimental design is then identified by the design matrix $\textrm{X}(s)$ in (4).

Extending the classical definition of the optimal design literature, see, for instance, (Atkinson et al. 2007), to the functional framework considered in this paper, an optimal experimental design is an experimental design $X^*(s)$ which minimizes a proper function Φ of the variance–covariance matrix of the estimator. From Proposition 1, it follows that an optimal design is given by

13 $$\begin{aligned} X^*(s)=\arg \min _{X(s) \in \mathcal C} \Phi (\mathrm {\Sigma } \otimes (\textrm{Z}^T \textrm{Z})^{-1}), \end{aligned}$$

with the *Z* given by (7) and C is the functional space where the factors are defined. Up to this point, we have maintained a general functional representation for the factors. However, to proceed with the design, it is necessary to express these factors using a basis expansion. The choice of basis functions defines the functional space, denoted by C, in which the factors reside. In practical applications, the selection of basis functions may be influenced by constraints arising from the underlying physics of the problem or from economic considerations. For instance, if the factors are required to be smooth, higher-order spline bases may be appropriate; if monotonicity is required, I-splines may be used (Ramsay 1988). In these cases, the constraints guide the selection of the basis functions directly. In other cases, the constraints must be incorporated into the optimization problem explicitly. Since such requirements are typically application-specific, we assume in the following that C is the space spanned by the selected basis functions, and that the optimization problem is unconstrained with respect to the basis coefficients. Nonetheless, it is straightforward to modify the formulation to include additional constraints by specifying certain coefficient values as inadmissible.

Let us expand each factor $\textbf{x}_i(s)$, for i=1,...,p, according to a proper basis $\{c^i_k(s), k \le 1\}$, and write the experimental conditions as finite linear combinations of the first $K_x^i$ elements of the respective basis:

$$\begin{aligned} x_{ni}(s)={\pmb {\gamma }_{n}^i}^T\, \pmb {c}^i(s), \end{aligned}$$

for any n=1,...,N and i=1,...,p, where ${\pmb {\gamma }_{n}^i}$ is a $K_x^i$-dimensional vector of coefficients.

Recalling that the model matrix (3) is obtained by adding a column of one’s to the matrix of the experimental conditions, we have that it can be rewritten as

$$\begin{aligned} \mathcal {X}(s)= \Gamma \textrm{C}(s), \end{aligned}$$

where

$$\begin{aligned} \textrm{C}(s)=\text{ diag }(1, \pmb {c}^1(s), \ldots ,\, \pmb {c}^p(s)), \end{aligned}$$

and Γ is a $N \times (1+ K_x^1+\cdots +K_x^p)$ block matrix given by

14 $$\begin{aligned} \Gamma =(\textbf{1}_N \,|\,\Gamma ^1 \, | \, \ldots \,| \, \Gamma ^p), \end{aligned}$$

with

$$\begin{aligned} \Gamma ^i=\begin{bmatrix} {\pmb {\gamma }_{n}^i}^T\\ \vdots \\ \\ {\pmb {\gamma }_{N}^i}^T \end{bmatrix} \end{aligned}$$

Hence, the matrix $\textrm{Z}$ in (7) can be rewritten as

15 $$\begin{aligned} \textrm{Z}= \Gamma \, \int \textrm{C}(s)\, \textrm{ H}(s)^T \,\textrm{d}s = \Gamma \, \textrm{J}_{CH} \end{aligned}$$

where

16 $$\begin{aligned} \textrm{J}_{CH}= \text{ diag }(1, \textrm{J}_{{c}{\eta }^1}, \dots , \textrm{J}_{{c}{\eta }^p}) \end{aligned}$$

and, for any i=1,⋯,p,

$$\begin{aligned} \textrm{J}_{{c}{\eta }^i}=\int \pmb {c}^i(s)\, \pmb {\eta }^i(s)^T\, \textrm{d}s. \end{aligned}$$

Hence, an optimal design $X^*(s)$ defined by Eq. (13) is identified by a matrix $\Gamma ^*$ such that

17 $$\begin{aligned} \Gamma ^*= \arg \min _{\Gamma } \Phi (\mathrm {\Sigma } \otimes ({\textrm{J}_{CH}}^T\Gamma ^T\, \Gamma \, \textrm{J}_{CH})^{-1}). \end{aligned}$$

### A- and D-optimality

In this section, we extend A- and D-optimality criteria to the dynamic experimental conditions: A functional A-optimal design $X^*_A(s)$ and a functional D-optimal design $X^*_D(s)$ satisfy the (13), with $\Phi ={{\,\textrm{tr}\,}}$, and $\Phi =\det$, respectively. Hence, the D- and the A-optimal designs ensure the precise estimation of the functional coefficients by minimizing the generalized variance and the average variance of the parameter estimators, respectively. In the following propositions, we derive the expression of these criteria.

#### Proposition 2

A functional A-optimal design $X^*_A(s)$ is obtained by minimizing

$$\begin{aligned} {{\,\textrm{tr}\,}}(\textrm{Z}^T \textrm{Z})^{-1}, \end{aligned}$$

where *Z* is the matrix defined in (7).

#### Proof

From Proposition 1 and from the property of Kronecker product that

$$\begin{aligned} {{\,\textrm{tr}\,}}(A \otimes B) = ({{\,\textrm{tr}\,}}A)( {{\,\textrm{tr}\,}}B), \end{aligned}$$

we have that

18 $$\begin{aligned} {{\,\textrm{tr}\,}}Var({{\,\textrm{vec}\,}}{\hat{\textrm{B}}})={{\,\textrm{tr}\,}}\Sigma \, {{\,\textrm{tr}\,}}(\textrm{Z}^T \textrm{Z})^{-1}; \end{aligned}$$

note that Σ is the covariance matrix of the error process and it is not affected by the design; then, if a functional design minimizes ${{\,\textrm{tr}\,}}(\textrm{Z}^T \textrm{Z})^{-1}$, it minimizes also the (18), and hence, it is A-optimal. □

#### Proposition 3

A functional D-optimal design $X^*_D(s)$ is obtained by maximizing

$$\begin{aligned} { \det (\textrm{Z}^T \textrm{Z}}), \end{aligned}$$

where *Z* is defined in (7).

#### Proof

Again, from Proposition 1 and from the property of Kronecker product that if A and B are an n×n and a m×m matrices, respectively, then

$$\begin{aligned} \det (A \otimes B)=(\det A)^m (\det B)^n, \end{aligned}$$

we have that

19 $$\begin{aligned} \det Var({{\,\textrm{vec}\,}}{\hat{\textrm{B}}}) =\dfrac{(\det \mathrm {\Sigma })^{1+\sum _{i=1}^p K^i}}{(\det \, \textrm{Z}^T \textrm{Z})^{L}}; \end{aligned}$$

then, if a functional design maximizes $\det \, \textrm{Z}^T \textrm{Z}$, it minimizes the (19), and hence, it is D-optimal. □

Propositions 2 and 3 show that that A-optimal and D-optimal designs do not depend on the unknown covariance matrix Σ of the error process. This is a crucial result because it allows us to generate optimal designs without the need of information from the data, which would be available only after the experiment.

Note that the expression therein contained is still based on the general representation of the design matrix, which is integrated to obtain the matrix *Z*. According to our method, the experimental conditions are expressed as linear combinations of proper basis of functions, and hence the optimization need to be done with respect to the basis coefficients, as it can be seen in equation (17). The optimal designs will then depend on the original choices of the bases for the factors and of the bases for the functional coefficients in the direction of *s*. The range of admissible functional shapes can be freely chosen by the experimenter, taking into account the experimental constraints. Several examples will be provided and discussed in Sect. 6.

## Results: optimal designs

This section is devoted to offer some examples of functional optimal experimental designs for precise estimation of the functional coefficient of model (3), based on the theory developed in the previous sections. The examples are organized as follows: First, we consider the same basis of functions to expand the functional coefficient and the runs of one functional predictor (Sect. 6.1); then, we extend to the case of choosing two different bases (Sect. 6.2); finally, we present an example with more than one predictor (Sect. 6.3).

In all of the examples, various B-splines (Smith 1980) are used as a choice for expanding the basis for both the functional factors and parameters. A B-spline is defined by its degree *D* and its knot vector. If we assume that the knot vector is constructed by equidistant points in the interval [0, 1], then we can define it in terms of the number of breakpoints $\mathcal {K}$ (in our notation including the two bound points 0 and 1). For example, the knots vector with $\mathcal {K}=5$ should be $\{0, 0.25, 0.5, 0.75, 1\}$. The length for a basis of degree *D*, using $\mathcal {K}$ knots in the knot vector, is $D + \mathcal {K} - 1$.

In the examples with one functional predictor, we assume that N=12 runs are available and we investigate how increasing the number of breakpoints for expanding the functional factor $\textbf{x}(s)$ affects the optimality. Additionally, we increase the number of break points in the expansion of β(s,t) with respect to *s*, to investigate how the design output chances for a constant number of breakpoints in *X*(*s*). For a design to be estimable, we need the total number of parameters (plus one to include a constant term according to (3)) to be less than the number of experimental runs. So if we expand β(s,t) with a B-spline of degree $D_\beta$ and $\mathcal {K}_\beta$ breakpoints, we need $N > D_\beta + \mathcal {K}_\beta$. Additionally, if we use a B-spline of degree $D_X$ and $\mathcal {K}_X$ knots for expanding the functional factor *X*(*s*), we need to enforce $D_X + \mathcal {K}_X \ge D_\beta + \mathcal {K}_\beta$, to ensure identifiability of (3).

Additionally, we explore scenarios involving multiple functional predictors to demonstrate how the interplay between different factors and their respective basis expansions influences the optimal design. Specifically, Sect. 6.3 presents an example with more than one predictor, illustrating the complexities and considerations that arise when extending the design framework to accommodate multiple functional inputs.

The algorithm used is a version of the coordinate exchange (Meyer and Nachtsheim 1995) using 1000 random starts implemented in Python 3.10 (Van Rossum and Drake 2009). The code is available by the authors upon request. The examples are referred to A-optimality, and similarly, D-optimum designs could be obtained.

### Same basis of functions to expand the dynamic factor and the parameter

In this scenario, we consider one functional factor and we expand both *X*(*s*) and the functional parameter β(s,t) using zero-degree B-spline basis functions ($D_X = 0$ and $D_\beta = 0$). Zero-degree B-splines are defined over a set of equidistant breakpoints (or knots) within the domain of the function. Between each pair of consecutive breakpoints, the basis function is constant.

In our analysis, we investigate how varying the number of breakpoints in the B-spline basis for *X*(*s*) affects the A-optimality criterion value while keeping the basis and number of breakpoints of β(s,t), with respect to the *s* variable, fixed. More specifically, we assume $\mathcal {K}_X \in \{3, 5, 9, 15, 19, 29\}$ and $\mathcal {K}_\beta \in \{3, 5,7\}$ and we vary the number of breakpoints $\mathcal {K}_X$ for each fixed $\mathcal {K}_\beta$.

Table 1 summarizes the A-optimality values and the relative efficiencies for different combinations of $\mathcal {K}_X$ and $\mathcal {K}_\beta$. The computed efficiency is relative to the best A-optimality value achieved in each case.

**Table 1:** A-optimality values and relative efficiencies for zero-degree B-splines expansion of *X*(*s*) and β(s,t) with varying numbers of breakpoints in *X*(*s*)

*Breaks*	*3*		*5*		*7*	
	A-opt	Efficiency	A-opt	Efficiency	A-opt	Efficiency
*3*	0.75	1.00	–	–	–	–
*5*	0.75	1.00	5.42	1.00	–	–
*9*	0.75	1.00	5.42	1.00	27.25	0.69
*15*	0.75	1.00	6.33	0.86	23.46	0.80
*19*	0.75	1.00	6.10	0.89	18.70	1.00
*29*	0.75	1.00	5.42	1.00	21.04	0.89

From Table 1, we observe that when $\mathcal {K}_\beta = 3$, the efficiency remains constant at 100% no matter the number of breakpoints for *X*(*s*). This indicates that increasing the number of breakpoints of the predictor’s basis functions does not improve the estimation precision of β(s,t) when the coefficient function is represented with only two ($D_\beta =0$, $\mathcal {K}_\beta =3$) basis functions.

**Fig. 2:**
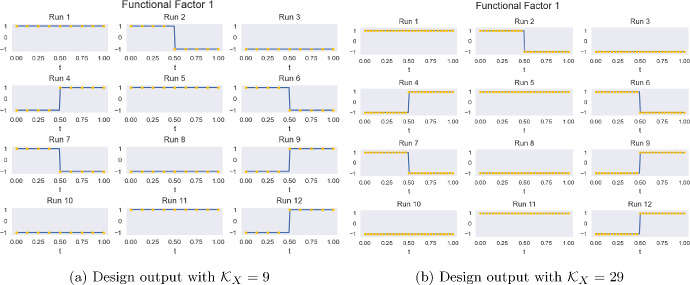
Comparison of design output for different breakpoints when $\mathcal {K}_\beta = 3$

This observation is further illustrated in Fig. 2, where we compare the optimal design outputs for $\mathcal {K}_X = 15$ and $\mathcal {K}_X = 29$ with $\mathcal {K}_\beta = 3$. Despite the increase in the number of breakpoints for *X*(*s*), the overall shape of the optimal design remains unchanged. Essentially, the simplicity of the coefficient function limits the complexity that can be captured in the design, and thus, even with more breakpoints, the optimal design does not change.

In contrast, when $\mathcal {K}_\beta = 7$, allowing for a more flexible representation of the factor by increasing the number of breakpoints improves the efficiency of the design, albeit with diminishing returns. A more flexible basis for *X*(*s*) allows the design to capture the increased complexity of the more intricate coefficient function β(s,t), enhancing the estimation precision.

**Fig. 3:**
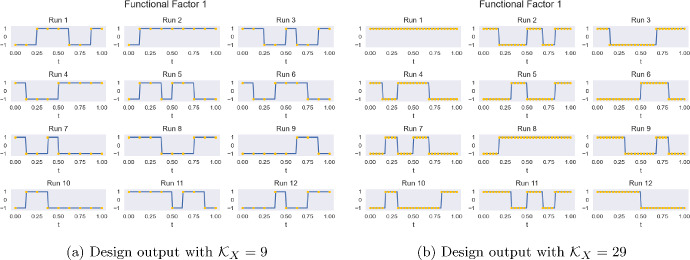
Comparison of design output for different breakpoints when $\mathcal {K}_\beta = 7$

This can be seen in the design output in Fig. 3. Increasing the number of breakpoints for *X*(*s*) from 9 to 29 changes the shapes. Upon closer inspection of Fig. 3, we see that there is no run in the left panel that matches in shape any from the right panel. This reinforces our belief that a more complicated expansion for the functional factor is better at capturing a more complicated expansion of the functional parameter.

An interesting observation arises from Table 1 concerning the case with 19 breakpoints for *X*(*s*) and 7 breakpoints for β(s,t). Notably, this specific configuration yields a better A-optimality criterion value than the case with 29 breakpoints for *X*(*s*). To understand this why this happens, it is essential to examine the knot vectors of *X*(*s*) and β(s,t) in this scenario.

We deduce that the knot vector of β(s,t) is included within the knot vector of *X*(*s*) when 19 breakpoints are used. This inclusion means that every knot of β(s,t) aligns with a knot of *X*(*s*), facilitating a more precise estimation of the coefficient function. In contrast, this alignment does not occur for *X*(*s*) with 9 or 15 breakpoints, where the step sizes do not result in such inclusion. This observation suggests that carefully aligning the knot vectors of *X*(*s*) and β(s,t) can enhance the estimation precision, and the knot vector of the predictor should be thoughtfully selected based on that of the coefficient function.

A similar rationale applies to the case with 5 breakpoints for β(s,t) and 15 or 19 breakpoints for *X*(*s*). In these scenarios, the knot vector of the coefficient function is not included within that of the predictor. As a result, the design cannot achieve 100% efficiency compared to cases where such inclusion exists.

These findings suggest the importance of matching not only the number of knots of the predictor’s basis functions with the complexity of the coefficient function but also ensuring the alignment of their knot vectors. When the coefficient function is more intricate, increasing the number of knots of the predictor’s basis functions is beneficial. However, optimal efficiency is further achieved when the knot vectors are carefully constructed so that the knots of the coefficient function are included within those of the predictor. This alignment allows the predictor to capture the essential features of the coefficient function more effectively, leading to improved estimation precision.

In the next section, we will explore scenarios where different families of basis functions are employed for the functional predictor and the parameter. This investigation will further elucidate how the choice of basis functions and the alignment of knot vectors impact the efficiency and effectiveness of the experimental design.

### Different bases of functions

In this scenario, we explore the optimal experimental design when the functional predictor *X*(*s*) and the functional coefficient β(s,t) are expanded using different bases functions. Specifically, we consider cases where the predictor and the coefficient function are represented using B-splines of different degrees. This investigation aims to understand how the choice of basis functions for the predictor and the coefficient affects the efficiency of the design.

We focus on two sub-scenarios:

1. $D_X=1$ and $D_\beta = 2$: This setup allows us to assess the impact of having a more complex coefficient function relative to the input factor.
2. $D_X=1$ and $D_\beta =0$: This contrast helps us understand the effect when the factor’s flexibility exceeds that of the coefficient function.

In both sub-scenarios, we vary the number of breakpoints (knots) in the B-spline basis functions for *X*(*s*) to observe how increasing the number of knots of the predictor influences the A-optimality criterion and the efficiency of the design.

#### First-degree B-splines for *X*(*s*) and second-degree B-splines for β(s,t)

In this sub-scenario, we investigate the optimal design when the functional predictor *X*(*s*) is expanded using first-degree B-spline basis functions (piecewise linear functions), and the coefficient function β(s,t) is expanded using second-degree B-spline basis functions (piecewise quadratic functions). This setup allows us to examine the effect of using a basis for the coefficient function that is allowed to change in more points relative to the predictor.

We consider varying the number of breakpoints in the B-spline basis for *X*(*s*) ($\mathcal {K}_X \in \{5,9,15,19,29\}$), while keeping the number of breakpoints for β(s,t) fixed at three different values as per the previous example ($\mathcal {K}_\beta \in \{3,5,7\}$). By analyzing both cases, we can assess how increasing the complexity of the coefficient impacts the efficiency of the design and precision of estimation when combined with predictor flexibility.

Table 2 summarizes the A-optimality values and relative efficiencies for these settings. The efficiency is calculated relative to the best A-optimality value achieved in each case, which corresponds to the maximum number of breakpoints for *X*(*s*).

**Table 2:** A-optimality values and relative efficiencies for first-degree B-splines expansion of *X*(*s*) and second-degree B-splines of β(s,t) with varying numbers of breakpoints in *X*(*s*)

*Breaks*	*3*		*5*		*7*	
	A-opt	Efficiency	A-opt	Efficiency	A-opt	Efficiency
*5*	36.82	0.60	–	–	–	–
*9*	23.71	0.92	112.84	0.71	352.12	0.60
*15*	22.49	0.98	84.44	0.95	256.99	0.79
*19*	22.19	0.99	83.93	0.96	216.76	0.94
*29*	21.96	1.00	80.30	1.00	207.83	1.00

**Fig. 4:**
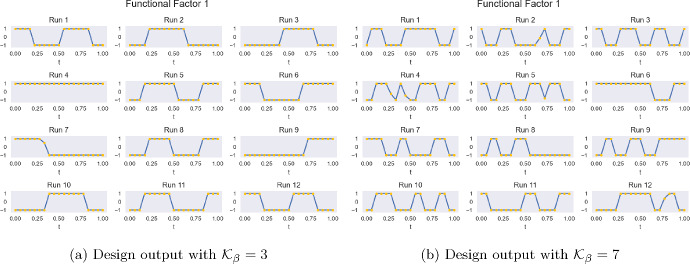
Comparison of design output for different breakpoints when $\mathcal {K}_X = 19$

In Fig. 4, we present two designs with a fixed number of breakpoints for *X*(*s*) ($\mathcal {K}_X = 19$), for $\mathcal {K}_\beta \in \{3,7\}$. We can observe that increasing the complexity of the functional parameter significantly impacts the design. The optimal design adapts by utilizing the available knots in *X*(*s*) to generate more varied predictor functions for the more complicated β(s,t). This is clear when comparing the changes in slope between Figs. 4a, and 4b. Fig. 4a shows much fewer changes in slope for each experimental run because the coefficient function is simpler.

This sub-scenario demonstrates that when the predictor’s representation is fixed, increasing the complexity of the coefficient function requires the design to make full use of the available knots to achieve optimal estimation. The designs become more intricate, with more changes in slope in the predictor functions, reflecting the need to capture the additional complexity in β(s,t).

#### First-degree B-splines for *X*(*s*) and zero-degree B-splines for β(s,t)

In this last sub-scenario, we explore the optimal design when the functional predictor *X*(*s*) is expanded using first-degree B-spline basis functions, while the coefficient function β(s,t) is expanded using zero-degree B-spline basis functions. This setup allows us to examine the effect of using a more flexible basis with respect to the number of breakpoints for the predictor relative to a simpler coefficient function.

We will again vary the number of breakpoints $\mathcal {K}_X$ in the B-spline basis for *X*(*s*) while keeping the number of breakpoints for β(s,t) fixed. We assess how the predictor’s number of knots impacts the efficiency of the design and the estimation precision when the coefficient function is relatively simple.

**Table 3:** A-optimality values and relative efficiencies for first-degree B-spline expansion of *X*(*s*) and zero-degree B-spline expansion of β(s,t) with varying numbers of breakpoints in *X*(*s*)

*Breaks*	*3*		*5*		*7*	
	A-opt	Efficiency	A-opt	Efficiency	A-opt	Efficiency
*2*	1.58	0.49	–	–	–	–
*3*	1.34	0.58	–	–	–	–
*5*	0.97	0.80	15.01	0.40	–	–
*9*	0.85	0.91	8.13	0.74	32.21	0.66
*15*	0.80	0.97	6.35	0.95	25.17	0.85
*19*	0.79	0.98	6.11	0.99	24.69	0.86
*29*	0.77	1.00	6.04	1.00	21.34	1.00

In Table 3, we again observe the same thing as with the previous sections. The efficiency of the design increases for all cases of $\mathcal {K}_\beta$ when we allow *X*(*s*) to vary between more breakpoints, with diminishing returns.

In scenario 6.1, we assumed the same zero-degree B-spline basis for the expansion of β(s,t) as in this scenario. Hence, the A-optimality values presented in Table 3 are directly comparable to Table 1 from scenario 6.1. The only thing that changed between those two scenarios is the type of basis function chosen for expanding the functional factor. In scenario 6.1, we used a zero-degree B-splines basis, and in this scenario, we allow for a more complex first-degree B-splines basis.

In Table 3, we can see that even at the maximum number of breakpoints, $\mathcal {K}_X=29$, we never quite reach the same A-optimality values as in Table 1 of scenario 6.1 with respect to $\mathcal {K}_\beta$. This suggests that the design with the more abrupt changes in slope (scenario 6.1) produces a more efficient design that the one with a more gradual change in slope (scenario 6.2.2).

**Fig. 5:**
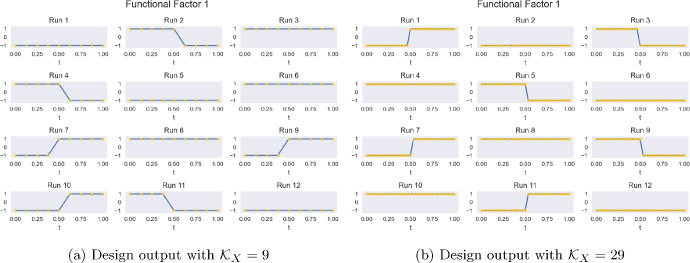
Comparison of design output for different breakpoints when $\mathcal {K}_\beta = 3$

To visualize the impact of the different basis functions, we present Fig. 5, which shows the optimal designs for $\mathcal {K}_X=9$, $\mathcal {K}_\beta =29$ and $\mathcal {K}_\beta =3$.

By comparing Figs. 2a to 5a, we see that the shapes of each experimental run of the functional factors are the same, given the constraints of each basis. For example, either the function experiences no change in slope and is pushed to the extreme values of the [-1,1] range that they are allowed to take, or they show one change in slope at the mid-point of the domain (0.5). The zero-degree B-spline basis of Fig. 2a results in more abrupt jumps from the one extreme point to the other, while the smoother first-degree B-spline basis of Fig. 5a has a more gentle slope going from one extreme to the other. The same can be said for the comparison between Figs. 2b and 5b.

This last sub-scenario demonstrates that increasing the predictor’s complexity by using higher-degree basis functions does not necessarily improve the estimation precision when the coefficient function is relatively simple. Overall, this analysis underscores that the choice of basis functions for the predictor and coefficient function should be carefully considered in functional experimental design.

### More than one predictor

In this final scenario, we extend our investigation to the case of multiple functional predictors. Specifically, we consider a model with two functional predictors $\textbf{x}_1$ and $\textbf{x}_2$, each one expanded using different B-spline basis functions. This example allows us to explore how the interplay between multiple predictors and their corresponding coefficient functions affects the optimal design and estimation precision.

The predictors and their associated coefficient functions are expanded as summarized in Table 4.

**Table 4:** Degree of the B-spline and number of breakpoints for the two functional factors and coefficient pairs

		*Component 1*		*Component 2*	
		Degree	Breakpoints	Degree	Breakpoints
Factor	$\textbf{x}(s)$	0	5	2	9
Coefficient	β(s,t)	0	3	1	3

In this scenario, we fix the number of breakpoints for both predictors. The A-optimality criterion value obtained for this design is 6.425.

**Fig. 6:**
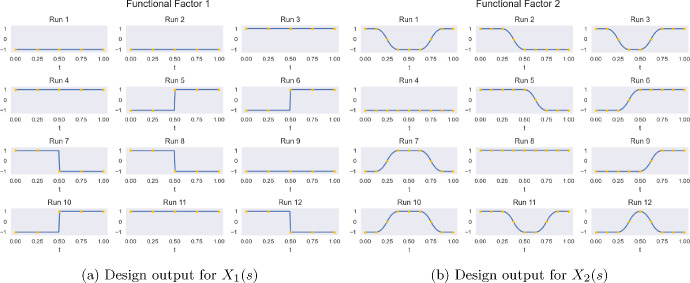
Design of experiments for two experimental factors

Figure 6 presents the optimal design for this scenario, displaying the experimental runs for both functional predictors $\textbf{x}_1(s)$ and $\textbf{x}_2(s)$. In this scenario, we observe that the design must accommodate for predictors with different degrees of the B-spline and number of knots, and their associated coefficient functions. The zero-degree B-spline expansion for $\textbf{x}_1(s)$ results in simpler experimental runs, suitable for estimating the relatively simple coefficient function $\beta _1(s,t)$. In contrast, the second-degree B-spline expansion for $\textbf{x}_2(s)$ provides the enough complexity to capture the intricacy of $\beta _2(s,t)$ which is expanded using first-degree B-splines.

Introducing multiple factors into a model increases the number of parameters that need to be estimated. In our experiments, we found that the coordinate exchange algorithm struggles to find an optimal design when the number of parameters is high. To address this issue, we can either expand the experiment by increasing the number of experimental runs or employ an alternative optimization algorithm.

In conclusion, the case of multiple functional predictors demonstrates that optimal experimental design can account for different complexities of the basis functions of each predictor and their associated coefficient functions. This example highlights that it is possible to accommodate the individual properties of each functional predictor and their associated coefficient functions in the design of experiments on the fully functional model.

## Proof-of-concept example

We develop a proof-of-concept example of our methodology from the continuous manufacturing line setting (Van Snick et al. 2019) described in Sect. 2. In each experimental run, a mixture is dosed into a gravimetric feeder (input), and the outlet concentration is monitored in-line downstream (output). Because of transport delays and mixing, sharp changes in the dosing program produce smoothed responses after a temporal lag. This naturally yields a function-on-function regression problem, where both factors and responses are trajectories on the normalized interval [0, 1].

**Simulation setup and data generation**

To generate data, we specify a bivariate coefficient surface β(s,t) on $[0,1]^2$ and simulate responses

$$ Y_j(t)\;=\;\int _0^1 X_j(s)\,\beta (s,t)\,\textrm{d}s\;+\;\varepsilon _j(t),\qquad j=1,\dots ,n, $$

where $X_j(\cdot )$ is the dosing profile of run *j* and $\varepsilon _j(\cdot )$ is a mean-zero functional noise process. The *true* coefficient is represented on a B-spline basis with 4×4 cubic splines and random coefficients. The error is a Gaussian process with radial-basis-function kernel and small variance ($\gamma =10^{-3}$, $\sigma ^2=10^{-2}$), represented on a Fourier basis with 81 terms. The choice of a rich, smooth basis for the noise ensures realistic smoothness and Dispersion of the response curves while keeping the signal-to-noise ratio moderate.

**Designs under comparison.**

We compare three different designs, each one with n=24 runs (the exact designs are provided in the Appendix and visualized there):

1. **Multivariate baseline (naïve approach):** we discretize the dosing profiles at 10 equally spaced segments and 7 different responses and apply a classical multivariate A-optimal design on the resulting 10-dimensional factor space. This design was generated using the software JMP (SAS 2024), treating the discretized values of X(·) as independent factors. This corresponds to approximating the functional input by a finite grid and designing the experiment as a standard multivariate linear model.
2. **Step–step (basis-aligned to physical actuation):** the dosing trajectories are represented on a degree-0 B-spline basis with 11 knots, yielding 10 stepwise segments over s∈[0,1]. The coefficient surface used in the design criterion is expressed on the same degree-0 B-spline basis (11 knots), and the response direction is represented on a 7-term Fourier basis. This setup produces A-optimal designs whose runs correspond to stepwise input profiles consistent with realistic feeder actuation.
3. **Spline–spline (smooth dosing):** the dosing trajectories are represented using degree-2 B-splines with 9 knots, yielding 8 interior segments and smoother functional inputs. In the design criterion, the coefficient surface is expressed using degree-3 B-splines with 5 knots in the factor direction, while the response direction is again represented on a 7-term Fourier basis. This basis choice yields A-optimal designs whose runs correspond to smooth dosing programs.

The first design serves as a baseline obtained by discretization and classical multivariate optimal design. The other two are purely functional A-optimal designs constructed directly in the appropriate functional basis, reflecting either stepwise or smooth actuation of the dosing mechanism.

**Fig. 7:**
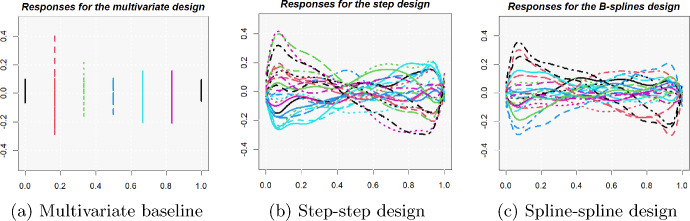
Simulated response curves for the three 24-run designs

Figure 7 displays typical responses produced by the three designs in a single simulated experiment. The multivariate baseline produces responses that are less diverse and exhibit—by design—blocking at the discretization points, whereas both basis-driven designs (step and B-spline) generate trajectories that explore the response space more effectively and more smoothly, better matching the expected physics of the process.

**Fig. 8:**
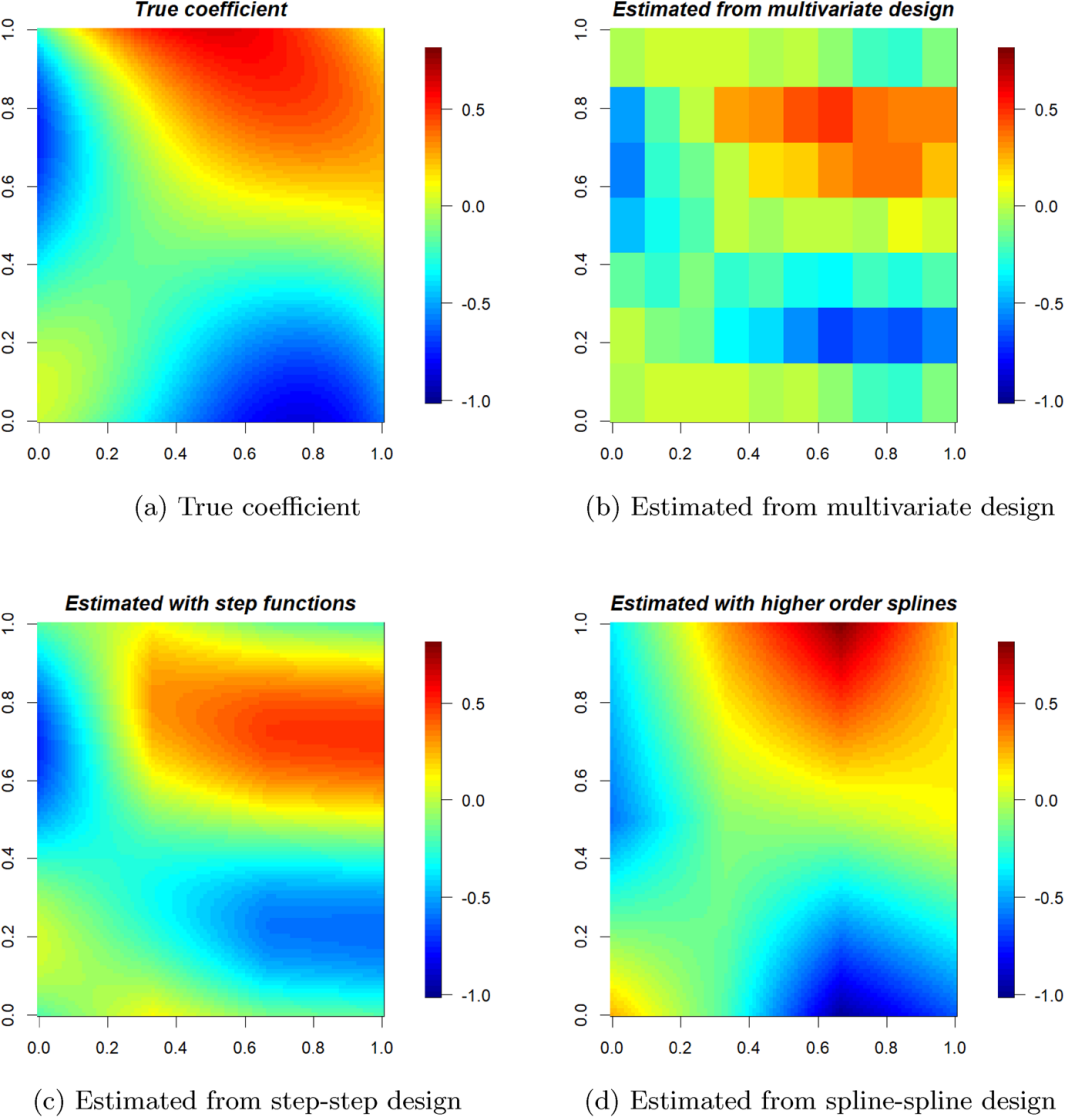
True coefficient and estimated coefficient surfaces under the three designs

The corresponding coefficient estimates are shown in Fig. 8. As expected, the multivariate fit yields a blocky estimate that misses smooth transitions, while the functional designs recover the main features of the true surface. Among them, the estimates produced by the spline–spline design are visually the closest to the true coefficient.

**Simulation study (RMSE).** To quantify the performances of the different designs, we repeated the simulation 100 times using the same true β(s,t) and error process and computed the RMSE on a 100×100 grid:

$$ \textrm{RMSE} \;=\; \biggl \{\frac{1}{10^4}\sum _{g,h}\bigl (\widehat{\beta }(s_g,t_h)-\beta (s_g,t_h)\bigr )^2\biggr \}^{1/2}. $$

**Fig. 9:**
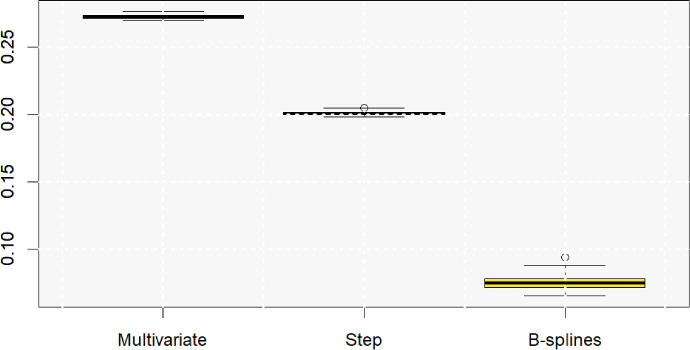
RMSE over 100 simulated experiments. Left to right: multivariate baseline, step–step, spline–spline

Figure 9 summarizes the distribution of RMSE across replicates. The spline–spline design attains the smallest errors, the step–step design is second, and the multivariate baseline is the worst. This ranking is consistent across repetitions and confirms that designing directly in function space yields more informative experiments than the naïve “discretize-then-design” approach.

These results demonstrate that functional A-optimal designs tailored to the structure of the factor and response spaces provide substantial gains over naïve, discretization-based, strategies. When the design space is expressed in an appropriate functional basis, capturing either the stepwise actuation of the process or its expected smoothness, the resulting experiments yield more informative response trajectories and hence, more accurate estimates of the coefficient surface.

## Conclusion

This work presents new results on optimal experimental design for estimating the functional coefficient of a linear model with multiple dynamic factors and where also the response is functional. The first part of the paper develops inference results and extends optimality criteria for selecting designs of experiments, proving that both A-optimal and D-optimality do not depend on the covariance of the error process.

The second part of the paper is dedicated to illustrating the proposed methodology in concrete examples. In order to construct an experimental design in this context, an experimenter should set in advance the set of basis functions (e.g., type, order, dimension, etc.) to be used for the predictors, and the set of basis functions to expand the functional coefficients in the direction of the predictors. As we have shown, the functional form of the optimal design is affected by this choice.

We describe then a real life example in an industrial scenario, and we apply our method with a simulation study. Simulations show that functional A-optimal designs provide substantial gains over naïve, discretization-based, strategies by yielding more informative response trajectories and hence, more accurate estimates of the coefficient surface.

The function-on-function model considered in this paper could be straightforwardly extended by including also non-functional factors and concurrent functional interactions. The extension to time-delayed interactions is more challenging, since the model includes functional coefficients in higher dimensions. This will be scope for future work. Moreover, in some cases it can be of interest to represent the functional coefficients using a higher number of basis functions and this can make the model not identifiable with the available number of runs. In this case, we need to include a penalization in the estimator, usually imposing a penalty on the derivatives of the functional coefficient. A further development will be to derive the expression of the optimal designs for this different estimator. Finally, other optimality design criteria, particularly those focusing on precise prediction, will also represent an interesting direction for additional development of the present work.

## Supplementary information

- An exhaustive database of the experimental designs generated in this paper is available online.^1^ The dataset consists of 576 records, each representing one experimental design with 12 columns detailing the settings and results. The columns include the design ID (id); constants such as the number of random starts of the coordinate exchange algorithm (epochs) and the number of experimental runs (Runs); choices made for the functional factor—basis type (X family), degree (X degree), and number of breakpoints (X breaks); and similar choices for the functional coefficient (B family, B degree, B breaks). The results comprise the design coefficients of the basis expansion of the functional factor, the A-optimality criterion value, and the figure of the design. If a design cannot be estimated for specific settings, the design column will have the value “NONE,” the criterion column will be null, and the figure column will contain no image.
- The three optimal designs constructed for Sect. 7 are showed in the Supplementary Material. This contains a table with the optimal multivariate design, a figure with the step function optimal design and a figure with the B-splines optimal design.

## Supplementary Material

We report the exact experimental designs used in the realistic proof-of-concept example discussed in Sect. 7 of the main paper. We provide (i) the 24-run multivariate A-optimal design used for the naïve discretize-then-design baseline and (ii) graphical representations of the corresponding 24-run functional A-optimal designs in the step–step and spline–spline settings. This material is intended to facilitate replication and to give additional insight into the structure of the proposed functional designs.

### Multivariate A-optimal design

Table 5 reports the 24-run multivariate A-optimal design (SAS 2024) employed for the baseline approach in Sect. 7 of the main paper. Each factor represents the value of the discretized input trajectory X(·) at one of ten equally spaced points on [0, 1], coded at levels -1 and 1. The resulting design is obtained by applying standard A-optimal design methodology to this 10-dimensional multivariate regression setting.

**Table 5:** Twenty-four-run A-optimal multivariate design used in the proof-of-concept study

Run	$X_1$	$X_2$	$X_3$	$X_4$	$X_5$	$X_6$	$X_7$	$X_8$	$X_9$	$X_{10}$
1	−1	−1	−1	−1	−1	−1	−1	−1	−1	−1
2	1	−1	−1	−1	−1	1	−1	1	−1	−1
3	1	1	−1	−1	−1	−1	1	−1	1	−1
4	1	1	1	−1	−1	−1	−1	1	−1	1
5	1	1	1	1	−1	−1	−1	−1	1	−1
6	−1	1	1	1	1	−1	−1	−1	−1	1
7	−1	−1	1	1	1	1	−1	−1	−1	−1
8	−1	−1	−1	1	1	1	1	−1	−1	−1
9	−1	−1	−1	−1	1	1	1	1	−1	−1
10	−1	−1	−1	−1	−1	1	1	1	1	−1
11	−1	−1	−1	−1	−1	−1	1	1	1	1
12	1	−1	−1	−1	−1	−1	−1	1	1	1
13	1	1	−1	−1	−1	−1	−1	−1	1	1
14	1	1	1	−1	−1	−1	−1	−1	−1	1
15	1	1	1	1	−1	−1	−1	−1	−1	−1
16	−1	1	1	1	1	−1	−1	−1	−1	−1
17	−1	−1	1	1	1	1	−1	−1	−1	−1
18	−1	−1	−1	1	1	1	1	−1	−1	−1
19	−1	−1	−1	−1	1	1	1	1	−1	−1
20	−1	−1	−1	−1	−1	1	1	1	1	−1
21	−1	−1	−1	−1	−1	−1	1	1	1	1
22	1	−1	−1	−1	−1	−1	−1	1	1	1
23	1	1	−1	−1	−1	−1	−1	−1	1	1
24	1	1	1	−1	−1	−1	−1	−1	−1	1

Each row corresponds to one experimental run, and each column $X_k$ to the coded level of the factor at the *k*-th discretization point in *s*

### Functional A-optimal designs

Figures 10 and 11 display the two functional A-optimal designs considered in the main paper, namely the step–step and spline–spline designs. In both cases, the input factor is a trajectory *X*(*s*) on [0, 1], corresponding to the dosing program applied to the gravimetric feeder. Each curve in the figures represents one of the 24 runs in the corresponding design.

Fig. 10 Twenty-four-run step–step A-optimal design. Each curve represents one dosing trajectory constructed using a step-function basis with ten segments over the normalized time domain s∈[0,1]

Fig. 11 Twenty-four-run spline–spline A-optimal design. Each trajectory is represented using second-order B-splines with ten segments over s∈[0,1]

#### Step–step design

In the step–step design, the input trajectories are constructed using a step-function basis with ten segments, reflecting a setting where the feeder operates at a finite number of discrete levels over successive time intervals. This basis aligns closely with realistic actuation patterns (on/off or few-level changes over time) and with the stepwise representation used when computing the A-optimal design in the factor space.

#### Spline–spline design

In the spline–spline design, the input trajectories are represented using second-order B-splines with ten segments, yielding smoother dosing profiles. This setting is appropriate when the actuation can vary more gradually over time, and it complements the use of smooth B-spline bases for both the factor and response directions in the estimation of the coefficient surface β(s,t).

## Data Availability

All the data used in the study were artificially created through simulations, and no real-world data were collected from experiments or observations. The optimal experimental designs are available via the supplementary material and the codes are available on request.

## Footnotes

1 https://airtable.com/appX72fWQTQ08lYkg/shrfeUlXNUS8kR2iV/tblLl0ckw7EVoaa7j.
