# Supplementary figures and images for: Optimal design of experiments for functional linear models with dynamic factors
Source: Test (Madr). 2026 Mar 12;35(2):469–97. doi: 10.1007/s11749-026-01004-z (PMC13427834; doi:10.1007/s11749-026-01004-z)

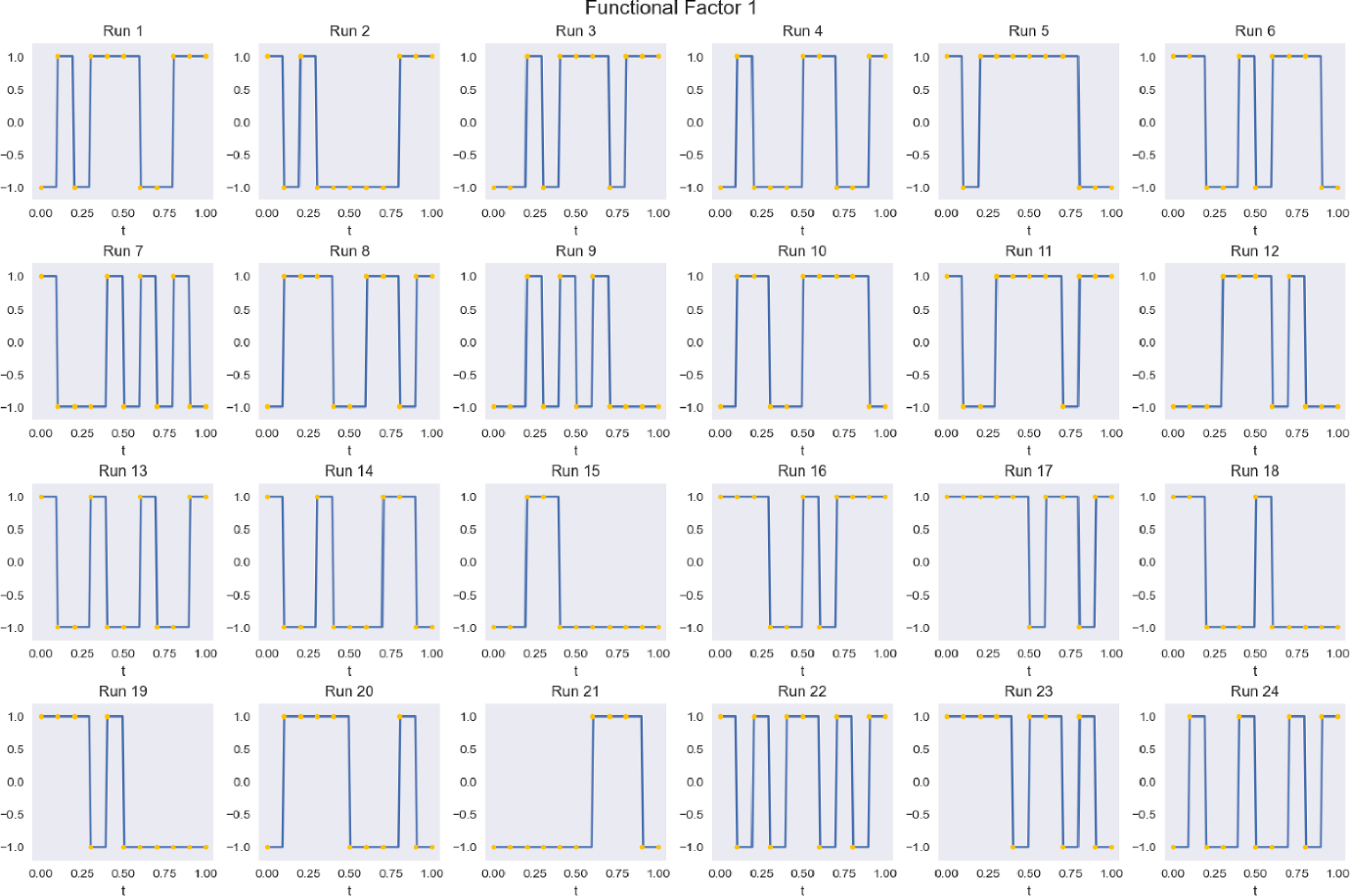

Supplement: Supplementary file 1 — Twenty-four-run step–step A-optimal design. Each curve represents one dosing trajectory constructed using a step-function basis with ten segments over the normalized time domain s ∈ [0,1]s∈[0,1] [file 11749_2026_1004_Fig10_HTML.png]

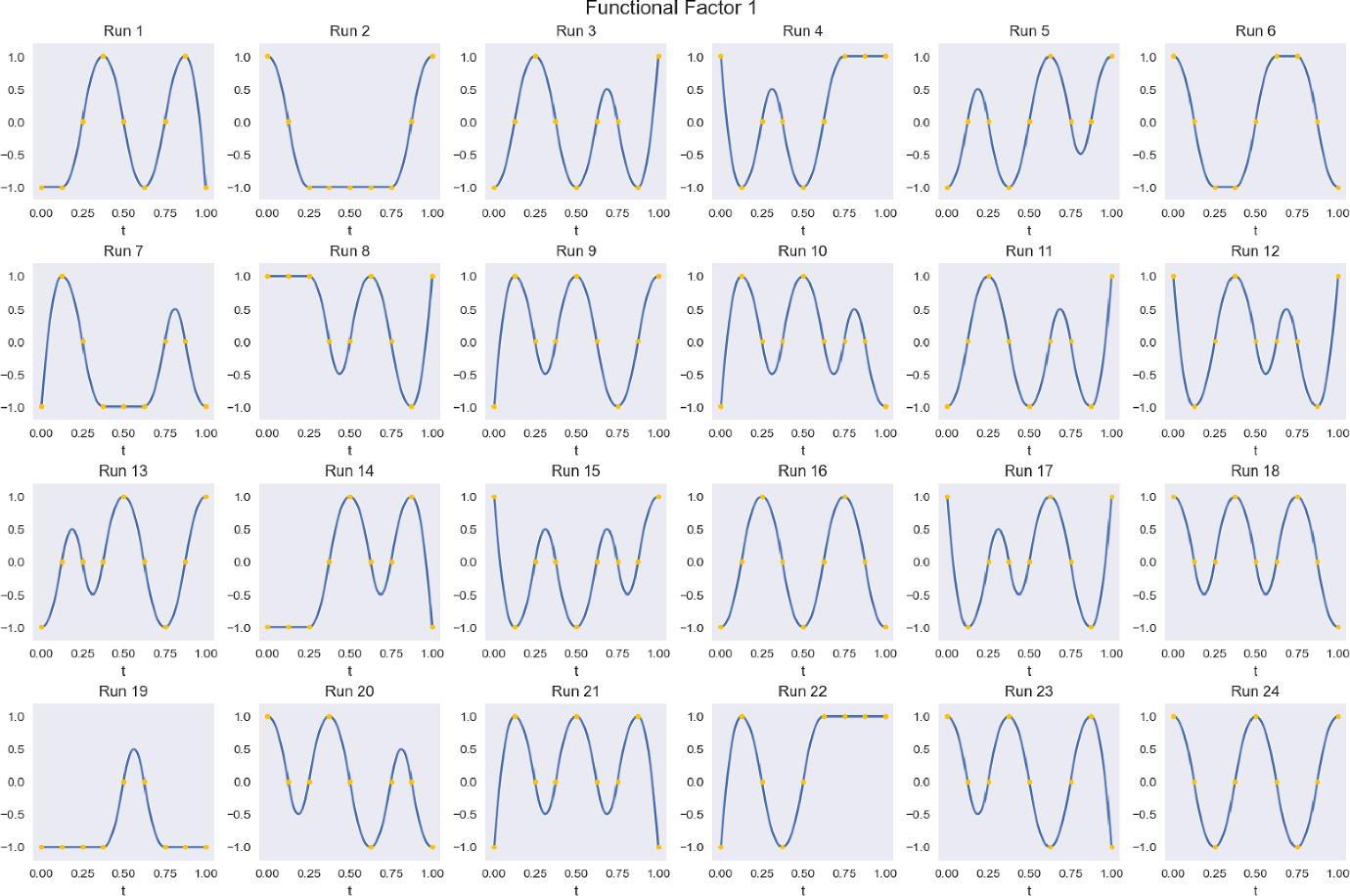

Supplement: Supplementary file 2 — Twenty-four-run spline–spline A-optimal design. Each trajectory is represented using second-order B-splines with ten segments over s ∈ [0,1]s∈[0,1] [file 11749_2026_1004_Fig11_HTML.png]
